# Supplementary material for: Enzymatic Synthesis of Vancomycin-Modified DNA
Source: Molecules. 2022 Dec 15;27(24):8927. doi: 10.3390/molecules27248927 (PMC9782525; doi:10.3390/molecules27248927)
Supplement: Supplementary file 1 [file molecules-27-08927-s001.zip › molecules-2056734-supplementary.pdf]

# Enzymatic synthesis of vancomycin-modified DNA

**Chiara Figazzolo [1,2], Frédéric Bonhomme [3], Saidbakhrom Saidjalolov [4], Mélanie Ethève-Quelquejeu [4] and Marcel Hollenstein [1,*]**

[1] Institut Pasteur, Université de Paris Cité, CNRS UMR3523, Department of Structural Biology and Chemistry, Laboratory for Bioorganic Chemistry of Nucleic Acids, 28, rue du Docteur Roux, CEDEX 15, 75724 Paris, France
[2] Learning Planet Institute, 8, Rue Charles V, 75004 Paris, France
[3] Institut Pasteur, Université de Paris Cité, Department of Structural Biology and Chemistry, Unité de Chimie Biologique Epigénétique, UMR CNRS 3523, 28 rue du Docteur Roux, CEDEX 15, 75724 Paris, France
[4] Université Paris Cité, CNRS, Laboratoire de Chimie et Biochimie Pharmacologiques et Toxicologiques, 75006 Paris, France
* Correspondence: marcel.hollenstein@pasteur.fr

## 1. Materials and methods

Solvents and reagents for the chemical reactions were obtained from Sigma-Aldrich, Alfa Aesar and Fisher Scientific. Flash chromatography was performed using silica gel (230-400 mesh) from Büchi on a Buchi Pure C-850 FlashPrep. HPLC purifications were performed using an Akta pure system (GE Healthcare) equipped with a DNA Pac PA 100 oligonucleotide column (250 X 4.0 mm) by Fisher scientific. MALDI-TOF experiments were performed on a Bruker ultrafleXtreme with 9-aminoacridine as a matrix in linear negative or reverse negative modes. High resolution electrospray ionization (ESI) mass spectra (MS, *m/z*) were recorded on a Waters Q-Tof Micro MS in the positive-ion electrospray ionization ($ESI^+$) mode. Solutions were prepared using 1:1 MeCN/$H_2O$ containing 0.1% formic acid. LC-MS/MS was performed on a Q exactive mass spectrometer (Thermo Fisher Scientific). It was equipped with an electrospray ionization source (H-ESI II Probe) coupled with an Ultimate 3000 RS HPLC (ThermoFisher Scientific) and with a ThermoFisher Hypersil Gold aQ chromatography column (100 X 2.1 mm, 1.9 µm particle size). DNA oligonucleotides used as templated and primers for PEX and PCR were purchased from Integrated DNA Technologies (IDT). All the DNA polymerases (Phusion, Therminator, Vent (*exo*$^-$), Deep Vent, Bst, Taq, Hemo Klen Taq, Phi29, Q5, Sulfolobus, and Klenow fragment of DNA polymerase I exo$^-$ (Kf (*exo*$^-$)), the natural dNTPs, the Monarch DNA Cleanup Columns (5 µg), the Nucleoside Digestion Mix, rSAP and Lambda (*exo*$^-$) were purchased from New England Biolabs (NEB). The agarose E-GELs and the E-GEL sample loading buffer (1X) was purchased from Fisher Scientific. PCR and PEX reactions were performed in a SimpliAmp Thermal Cycler by Applied Biosystems. The melting experiments were performed on an Agilent Cary UV-Vis 3500 Compact Peltier machine in 100 µL volume quartz cuvettes. Visualization of PAGE gels was performed by Fluorescence imaging using a Typhoon Trio phosphorimager from GE Healthcare. PCR Cleanup NucleoSpin columns and the Mini kit for plasmid DNA were purchased from Macherey-Nagel. The TOPO TA Cloning Kit for Sequencing was purchased by Invitrogen. The plasmid sequencing was performed by Eurofins Genomics.

## 2. Additional experimental protocols

General protocol for PCR with modified nucleotides

The primers (6 μM each), the template (0.1 μM), the natural and/or modified nucleotides (200 μM), $Mg^{2+}$ (2 mM), the polymerase (1 μL) and the appropriate polymerase buffer (2.5 μL of 10X or 5 μL of 5X) were mixed together in a total volume of 25 μL. Each reaction in presence of Vent (*exo*$^-$) or Deep Vent polymerases was characterized by 5 min 95 °C, 30 cycles composed by 30'' 95 °C, 30'' 57 °C, 2 min 72 °C, and a final extension step of 5 min 72 °C. Each reaction in presence of Phusion polymerase was characterized by 30'' 98 °C, 30 cycles composed by 10'' 98 °C, 30'' 72 °C, 2 min 72 °C, and a final extension step of 5 min 72 °C. The PCR products were analyzed by agarose gels (2%) with E-GEL sample loading buffer (1X).

Protocol for conversion of modified dsDNA into natural dsDNA *via* PCR

The modified dsDNA obtained *via* PCR was purified with PCR Cleanup NucleoSpin columns following the reported PCR Cleanup protocol and quantified. The purified products were applied as a template (10 nM) and mixed with the primers (500 nM), the dNTPs (200 μM), $Mg^{2+}$ (2 mM), the polymerase Vent (*exo*$^-$) (1 μL) and Thermopol buffer (3 μL of 10X) in a total volume of 30 μL. The PCR program was characterized by 5 min 95 °C, 10 cycles composed by 30'' 95 °C, 30'' 52 °C, 30'' 72 °C, and a final extension step of 5 min 72 °C. The PCR products were analyzed by agarose gels (2%) with E-GEL sample loading buffer (1X).

Protocol for cloning and sequencing

Cloning was performed using the TOPO TA Cloning Kit for Sequencing and following the relative published protocol. $dU^{Van}$-modified PCR products were purified and converted into natural dsDNA as described above using Taq as a polymerase. The PCR products (2 ng), the TOPO vector (10 ng) and the salt solution (200 mM NaCl, 10 mM $MgCl_2$) were mixed gently in an Eppendorf tube for a total volume of 6 μL and incubated for 5 minutes at r.t to form the TOPO Cloning reaction. Afterwards, the tube was transferred on ice. The TOPO Cloning reaction (2 μL) was added into a vial of One Shot TOP10 chemically competent *E. Coli* (provided with the Kit) and mixed gently. The mixture was incubated for 30 minutes on ice. The cells were heat-shocked at 42°C for 30'' without shaking and then immediately transferred together with their tube on ice. S.O.C. medium (250 μL) (provided with the Kit) at r.t. was added to the cells tube. The tube capped tightly and incubated horizontally for 1 hour at 37 °C shaking at 200 rpm. The transformation (50 μL) was spread on a pre-warmed LB plate containing 50 μg/mL ampicillin and 20 mg/mL X-Gal in DMF (40 μL). The plate was incubated overnight at 37 °C. To verify the correct incorporation of the DNA of interest in the TOPO plasmid and its cloning in the colonies, 19 white colonies were captured, and their DNA used as a template in the protocol 'General protocol for conversion of modified dsDNA in natural dsDNA *via* PCR' (successful results reported on a 2% agarose gel in Figure S12). After successful verification of the products, the plasmids of 15 of these colonies were purified using the Mini kit for plasmid DNA. The products were quantified *via* UV absorbance and nine were sent for Sanger sequencing to Eurofins Genomics.

Protocol for the generation of dU$^{Van}$-modified ssDNA

The protocol started with performing PEX according to the 'Protocol for verifying the incorporation of one dU$^{Van}$TP in dsDNA' in presence of a phosphorylated template. Afterwards, the products were purified by means of Monarch DNA Cleanup Columns (5 μg) (250 pmol product per column). The dU$^{Van}$-modified dsDNA (100 pmol) was incubated with λ-exonuclease (2.7 μL), Thermopol buffer (5 μL for 10X) in a total volume of 50 μL for 1.5 hours at 37 °C. The enzyme was then deactivated for 10 minutes at 75 °C. The nucleic acid solution (50 μL) was then mixed with an equal volume of phenol : chloroform : isoamyl alcohol (25:24:1) by pipetting for 20'' until formation of an emulsion. The emulsion was centrifuged at 12000 rpm for 5 mins at r.t. and a clear separation of the aqueous and organic phases was detected. The aqueous phase was carefully transferred in a clean Eppendorf tube, which was filled with ice cold EtOH previously incubated at -20 °C overnight. The tube was incubated at -80 °C for 1 hour and then centrifuged for 30 minutes at maximum speed at 4 °C. The EtOH was removed and the precipitate was washed with cold 70% EtOH. The remaining EtOH was evaporated iby exposure to air and the ssDNA was resuspended in $H_2O$ for UV absorbance quantification.

## 3. Chemical characterization of nucleosides and nucleotides

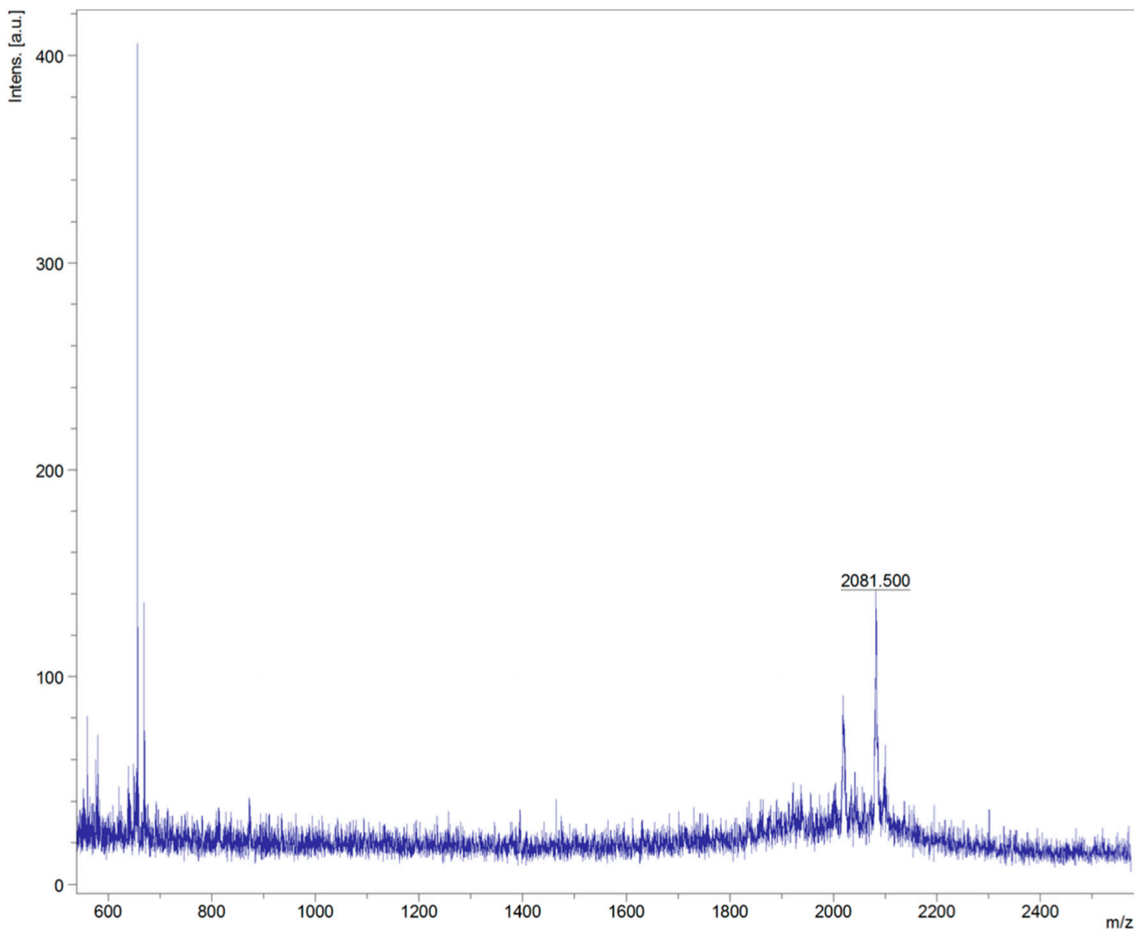


**Figure S1.** MALDI-TOF analysis of dU$^{Van}$TP coordinated with $Cu^{2+}$ in linear negative mode (0.7 KDa – 3 KDa, 65%).

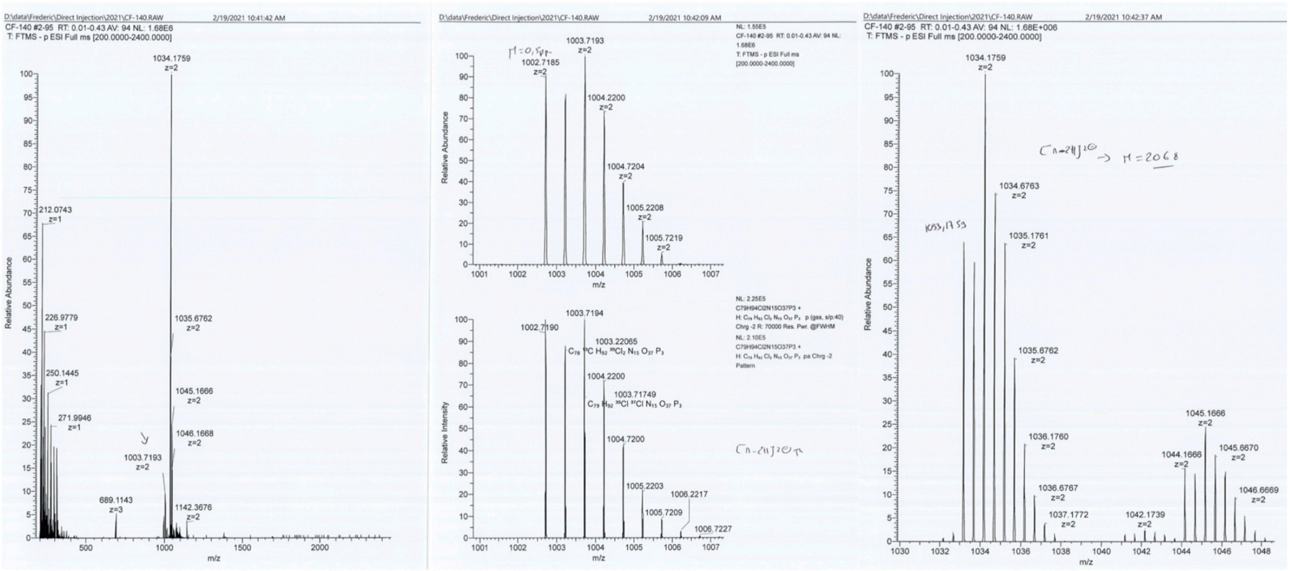

**Figure S2.** HR-ESI-MS analysis of dU$^{Van}$TP coordinated with $Cu^{2+}$. The uncoordinated dU$^{Van}$TP is also detected in this analysis ($m/z$ = 1034.1759 for $z$ = 2).

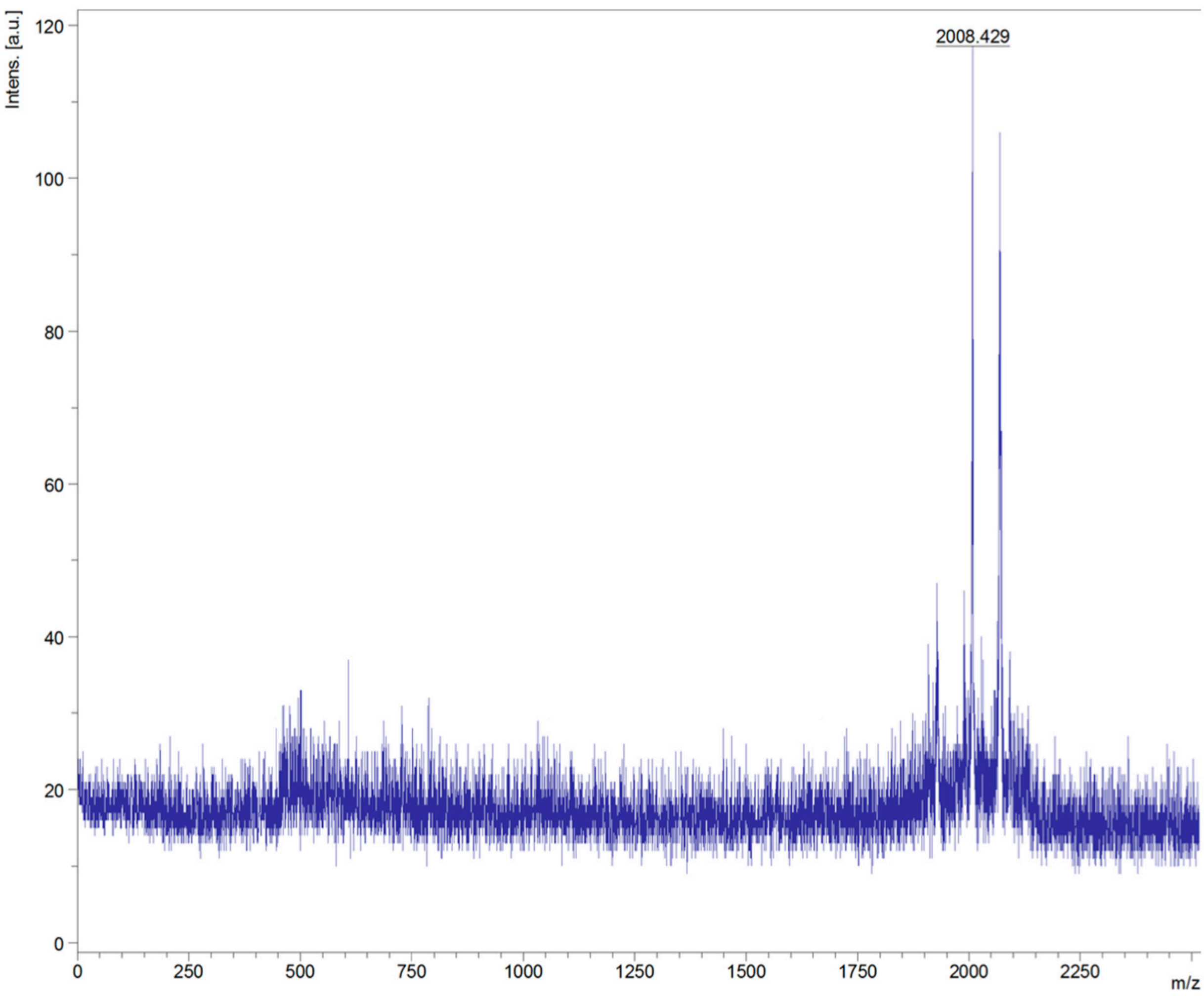


**Figure S3.** MALDI-TOF analysis of dU$^{Van}$TP in linear negative mode (0.7 KDa – 3 KDa, 65%).

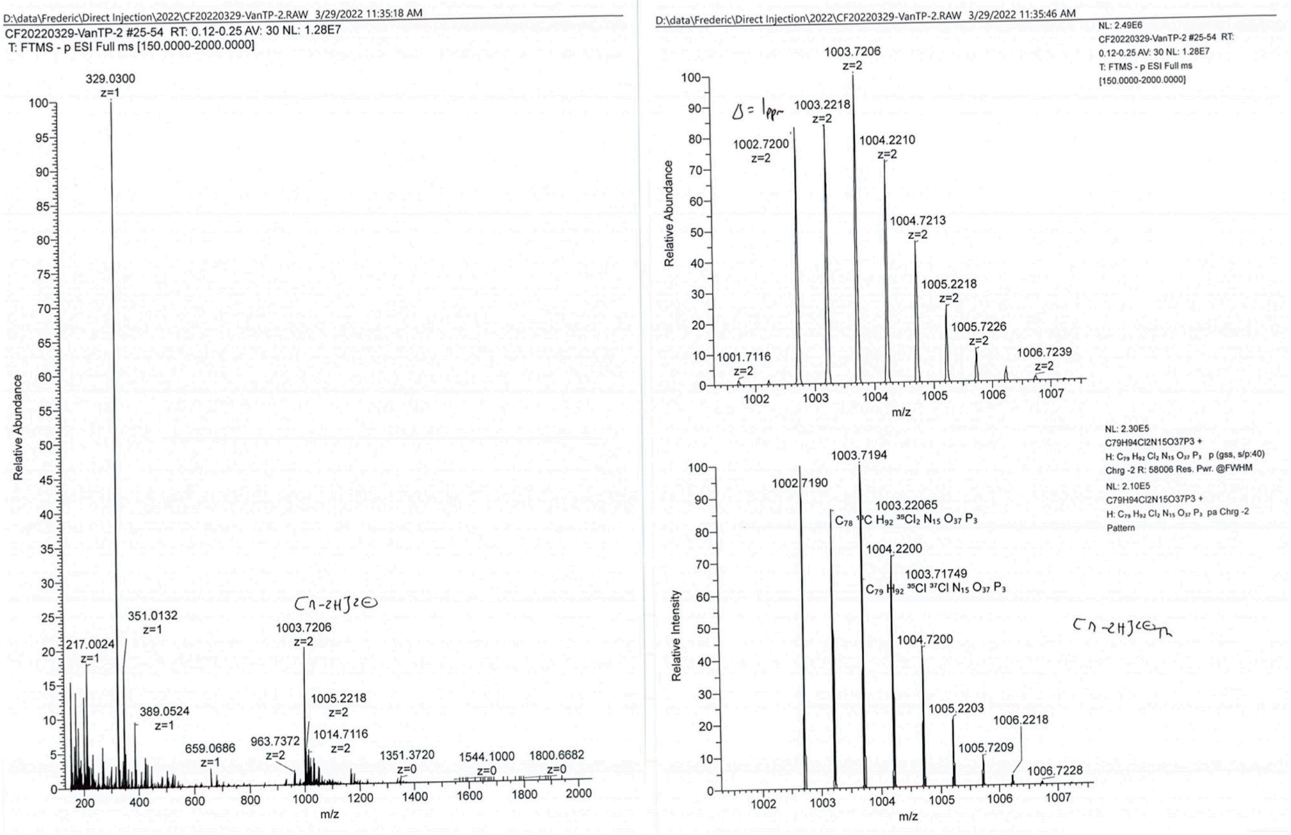

**Figure S4.** HR-ESI-MS analysis of dU$^{Van}$TP.

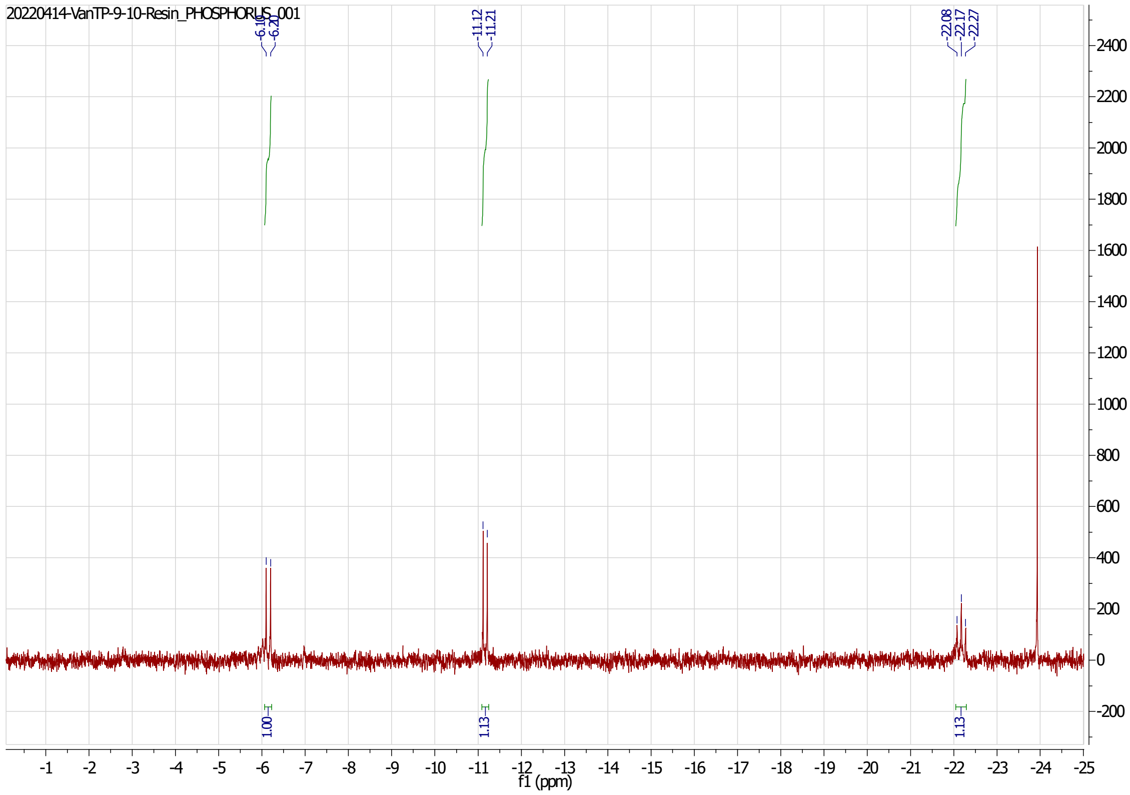

**Figure S5.** $^{31}$P NMR (202.4 MHz, $D_2O$) spectrum of dU$^{Van}$TP.

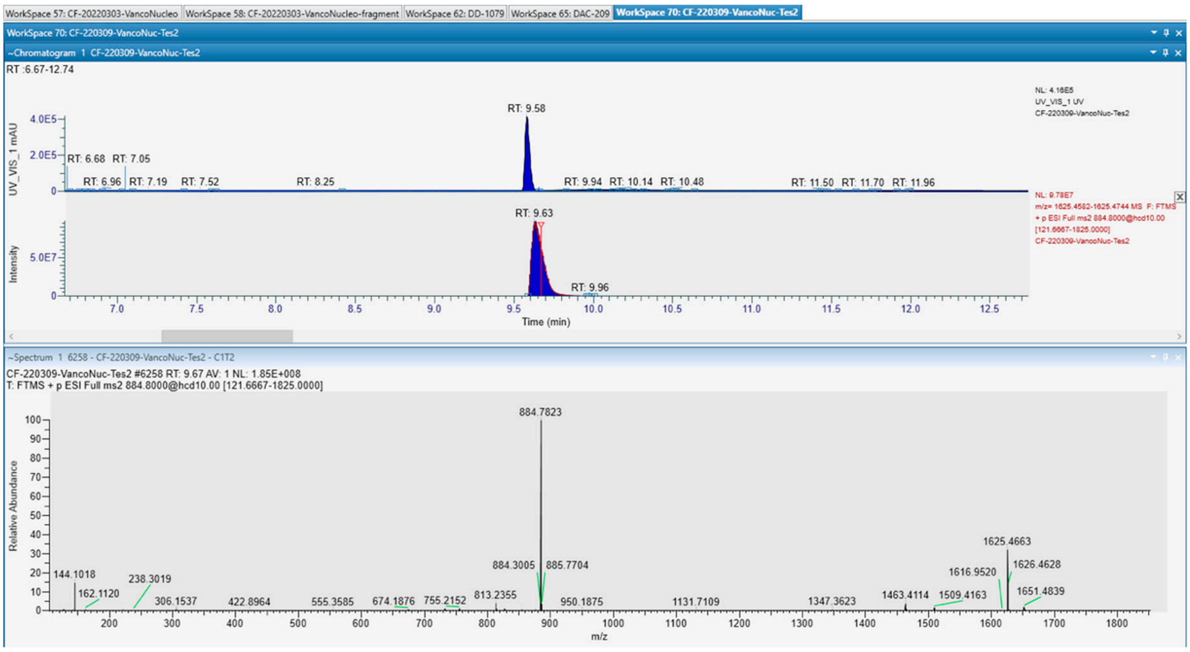


**Figure S6**. LC-MS analysis of dU$^{Van}$.

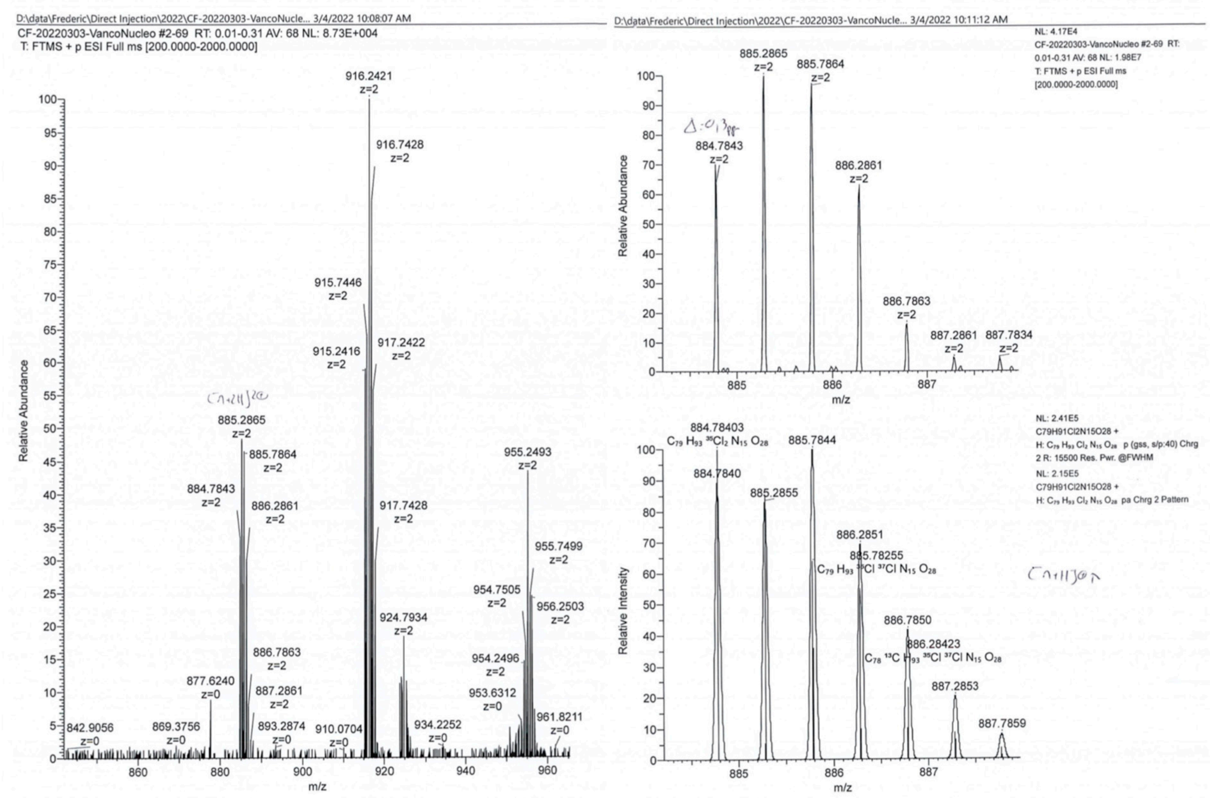


**Figure S7.** HR-ESI-MS analysis of nucleoside dU$^{Van}$.

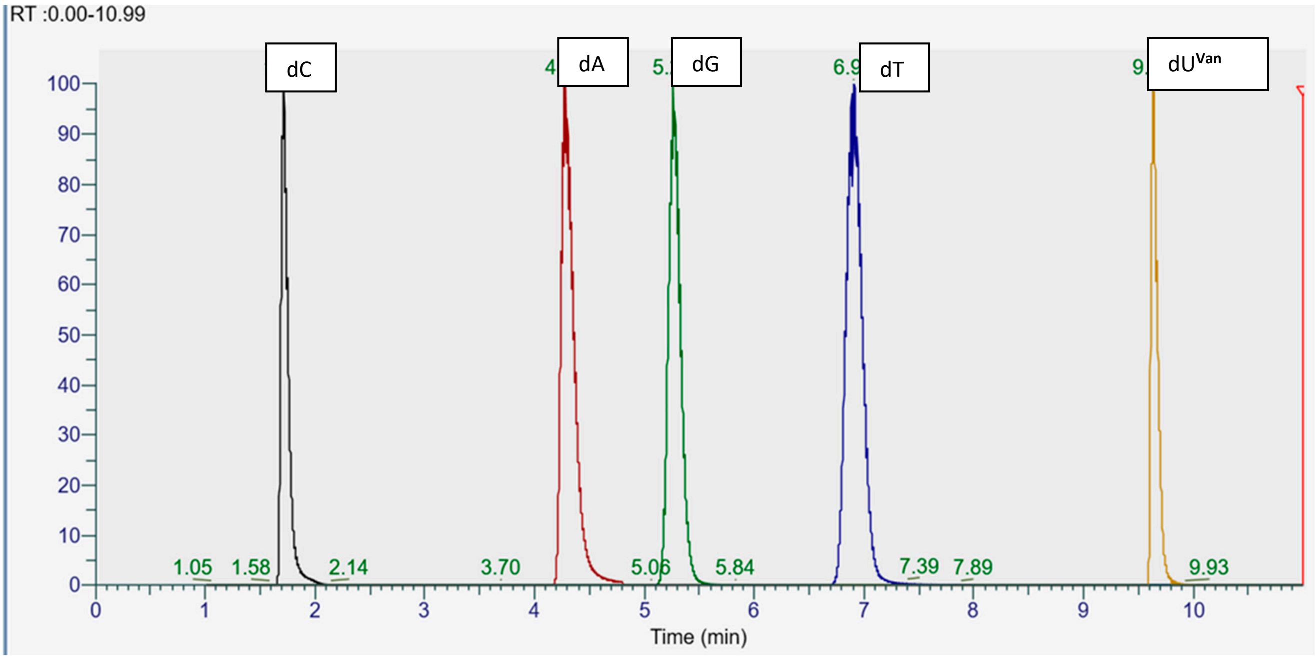


**Figure S8**. Full LC-MS chromatogram of the nucleosides stemming from the digested and dephosphorylated PEX reaction product obtained with $dU^{Van}TP$. In black: dC (MS/MS signal for the parent ion : 228.1 Da and fragment ion : 112.0506 Da); in red: dA (MS/MS signal for the parent ion : 252,1 Da and fragment ion : 136,0616 Da); in green: dG (MS/MS signal for the parent ion : 268.1 Da and fragment ion: 152,0565 Da); in blue: dT (MS/MS signal for the parent ion : 243.1 Da and fragment ion: 127,0501 Da); in mustard : $dU^{Van}$ (MS/MS signal for the parent ion : 884,8 Da ($z$=2) and fragment ion : 1625,4652 Da).

**4. Additional gel images**

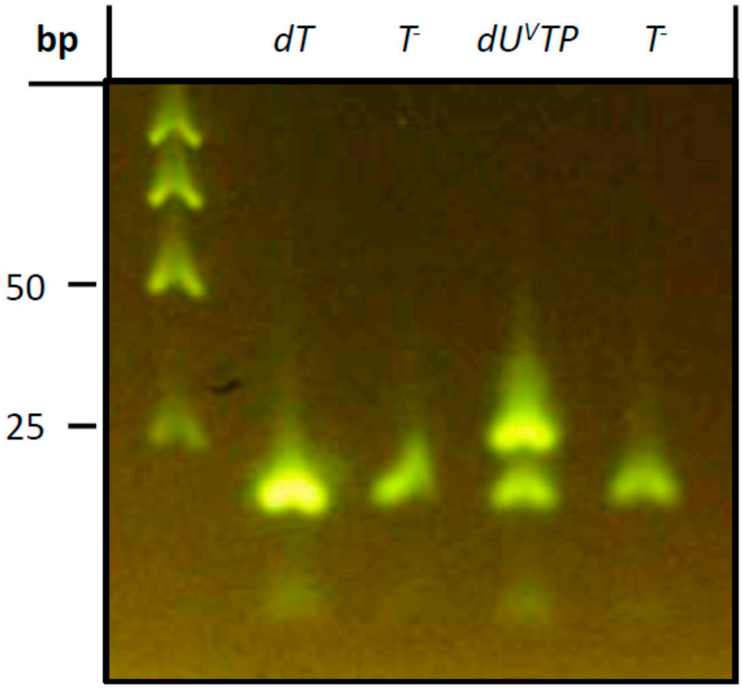


**Figure S9**. Gel image (agarose 4%) analysis of PEX reaction products obtained with dU$^{Van}$TP and dTTP. Each PEX reaction was carried out using primer **P1** (100 pmol), template **T1** (150 pmol), dU$^{Van}$TP/dTTP (200 μM), Thermopol buffer 10x (1 μL) and Vent (*exo*$^{-}$) polymerase (4U) in a total volume of 10 μL. The reaction mixtures were incubated at 60°C for 4 hours. Lanes from left to right: incorporation of one dTTP (lane dT), the negative control with no dTTP (T$^{-}$) , reaction product obtained with dU$^{Van}$TP (dU$^{V}$TP), the negative control without dU$^{Van}$TP (T$^{-}$).

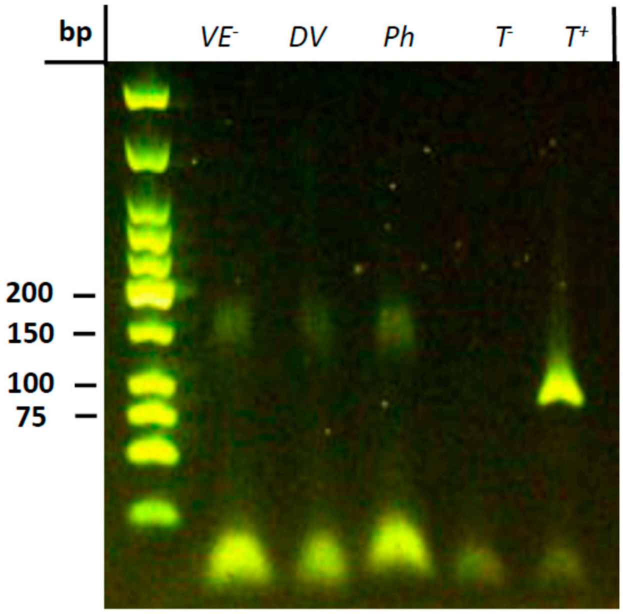


**Figure S10**. Gel image (agarose 2%) analysis of PCR reaction products obtained with dU$^{Van}$TP and dTTP. For reactions, Vent (exo⁻) (VE⁻) (2U), Deep Vent (DV) (2U) and Phusion (Ph) (2U) DNA polymerases were used with primers **P3** and **P4** and template **T2**. Each reaction was performed in 25 µL reaction volume and contained **T2** (0.1 nM), **P3**/**P4** (6 µL), dATP, dCTP, dGTP (200 µM), dU$^{Van}$TP (200 µM), $Mg^{2+}$ (2 mM), polymerase buffer (2.5 µL of 10X or 5 µL of 5X) and polymerase (1 µL). The negative control was performed without polymerase ($T^-$) and the positive control ($T^+$) contained dTTP instead of dU$^{Van}$TP. The PCR program for Vent (exo⁻) and Deep Vent was 95°C 5 minutes (min), (95°C 30'', 57°C 30'', 75°C 2 min) for 25 cycles, 75°C 5 min. The PCR program for Phusion was 98°C 30'', (98°C 10'', 72°C 30'', 72°C 1 min) for 25 cycles, 75°C, 5 min.

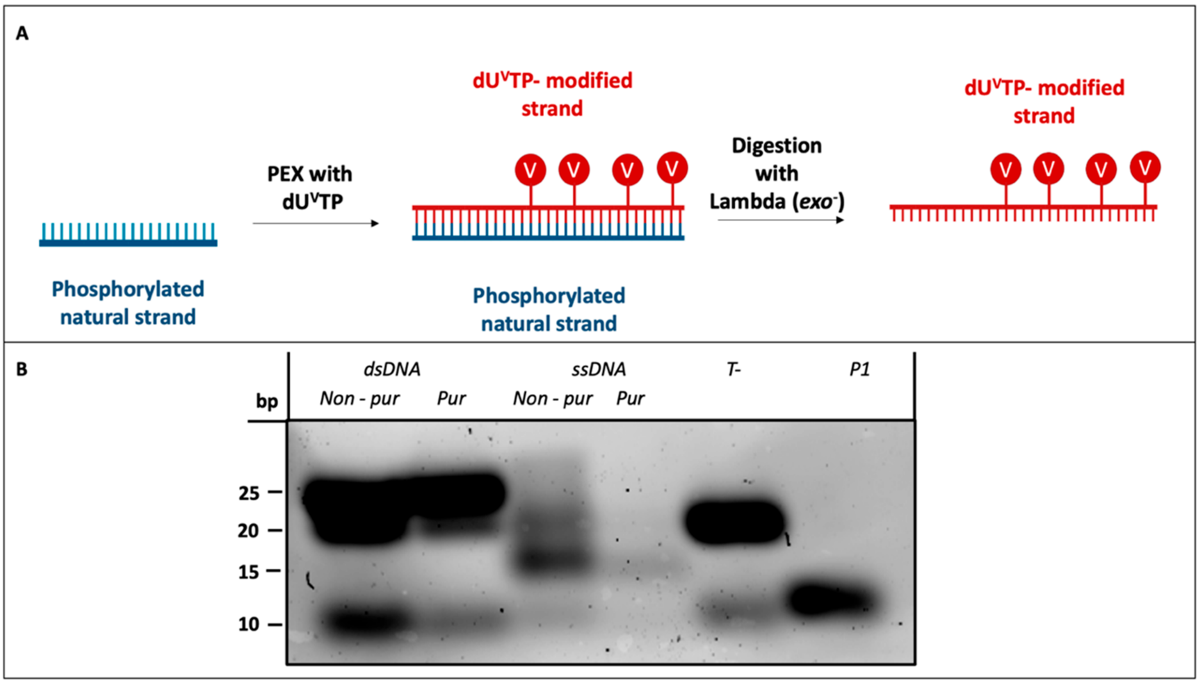


**Figure S11.** A) Schematic representation of the different steps involved in the generation of dU$^{Van}$-modified ssDNA from the corresponding dsDNA. Briefly, PEX reaction is performed on a 5'-phosphorylated strand in presence of a natural primer, dU$^{Van}$TP and the appropriate polymerase. Afterwards, the ssDNA is generated by digestion of the 5'-phosphorylated strand with λ-exonuclease. B) Gel image (agarose 4%) analysis of the products of the generation of the dU$^{Van}$-modified ssDNA. From left to right: modified dsDNA obtained from PEX reaction without purification; modified dsDNA after PEX reaction and purification; modified ss-DNA without further purification; modified ss-DNA after purification. The negative control T$^-$ was obtained by reproducing the PEX system without polymerase and **P1** corresponds to unreacted primer **P1**.

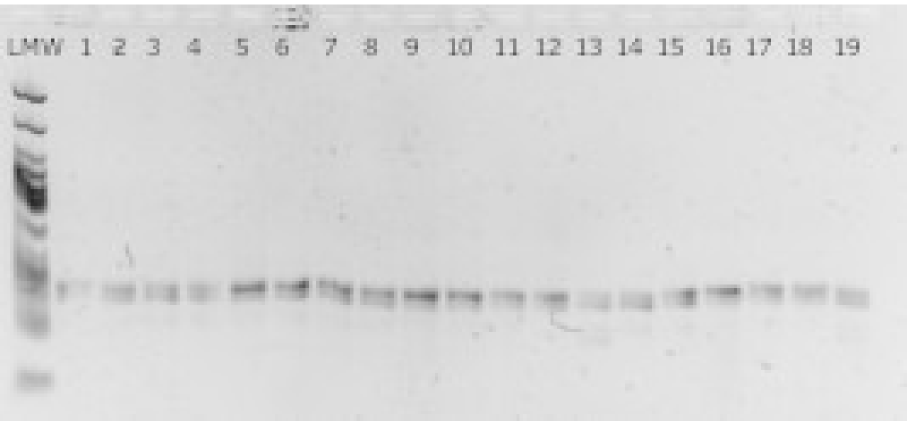


**Figure S12.** Gel image (2% agarose) showing the results obtained by performing PCR on 19 different *E. Coli* colonies. Conditions: **P3** and **P4** (500 nmol), dNTPs (200 μM), Mg$^{2+}$ (2 mM), Taq buffer (2.5 μL of 10X), Taq polymerase (5U) in 50 μL. PCR program: 5 min 95°C, (30'' 95°C, 30'' 52°C, 30'' 72°C) X 10 cycles, 5 min 72 °C.

## 5. Python script for data analysis of UV-melting experiments

The absorbance values and their associated temperatures for each heating ramp of the UV-melting experiments were converted into a dataframe. Afterwards, the absorbance values were normalized by conversion to hyperchromicity. To do so, we introduced a function that captures the minimum absorbance value in each heating ramp, and then divides every absorbance value of the ramp by the respective minimum value. The completion of the calculation led us to plot the hyperchromicity as a function of temperature for each ramp, and to visualize the results in a sigmoidal curve. After obtaining the hyperchromicities for each ramp, we applied a function that calculates for each hyperchromicity the value of its first derivative. We plotted the results in a Gaussian curve reporting the first derivative as a function of the temperature. Afterwards, we applied to the first derivative data a function that, given a set of data in input, gives as an output the values of the parameters of the Gaussian function that optimally fits the given data. When having the parameters of the curve, we plotted it together with the experimental data. Given the theoretical optimal curve for our experimental data, we used Python to calculate the theoretical absorbance value associated to each recorded temperature using the theoretical Gaussian curve. Then, we applied a Python function to these data that finds the maximum value in a set of data. In this way we found the maximum value of the absorbance for the theoretical curve, and we extrapolated the associated temperature value, corresponding to the $T_m$. This series of operations were performed for each heating ramp and, to calculate the final $T_m$, we did the average of the melting temperatures for each ramp. Moreover, from the average, we extrapolated the standard deviation.

```
import numpy as np
import pandas as pd
import matplotlib.pyplot as plt
import math
from scipy.optimize import curve_fit
from numpy import diff
```

```
'''
Before executing the code make sure:
    1) The Excel file with the original data has the correct header used in every Tm file
    2) The default first two lines have been cancelled
    3) All the values are in number format
    4) The decimal separator is .
    5) The Excel file is converted in Macintosh .csv in special formats
'''

Df_TmData = pd.read_csv('Vancomycin_Tm.csv', sep = ';')
```

```
Df_V = Df_TmData[['V1TH', 'V1AH', 'V2TH', 'V2AH', 'V3TH', 'V3AH']]
```

```
#The following line safely disables the warnings for chained assignements
pd.options.mode.chained_assignment = None

#For each heating ramp the hyperchromicity is calculated and added to the dataframe
Df_V['HyperCV1'] = Df_V['V1AH'].apply(lambda x: (((x-(Df_V['V1AH'].min()))/(Df_V['V1AH'].min()))*100))
Df_V['HyperCV2'] = Df_V['V2AH'].apply(lambda x: (((x-(Df_V['V2AH'].min()))/(Df_V['V2AH'].min()))*100))
Df_V['HyperCV3'] = Df_V['V3AH'].apply(lambda x: (((x-(Df_V['V3AH'].min()))/(Df_V['V3AH'].min()))*100))
```

```
#Ramp 1
Df_V.plot.scatter(x = 'V1TH', y = 'HyperCV1')
plt.xlabel('Temperature °C')
plt.ylabel('Hyperchromicity')
plt.title('1st Heating ramp Vancomycin')

#Find first derivative experimental point
Df_V['dHV1'] = Df_V['HyperCV1'].diff()
Df_V['dHV1'][0] = 0
Df_V.plot.scatter(x = 'V1TH', y = 'dHV1')

# Function to calculate the Gaussian with constants a, b, and c
def gaussian(x, a, b, c):
    return a*np.exp(-np.power(x - b, 2)/(2*np.power(c, 2)))

# Fit the Gaussian data
pars, cov = curve_fit(f=gaussian, xdata = Df_V['V1TH'], ydata = Df_V['dHV1'], p0 = [2.5, 63, 1], bounds=(-np.inf, np.
print(pars)
plt.plot(Df_V['V1TH'], gaussian(Df_V['V1TH'], *pars))

#Create list of values from 20 to 95 separated by 0.01 representing the temperatures
Temperature = []
x = 20
while x <= 95:
    Temperature.append(x)
    x+=0.01

#Calculate the y value for each x based on the Gaussian equation found
a = pars[0]
b = pars[1]
c = pars[2]
Derivatives = gaussian(Temperature, a, b, c)
DF_derivatives = pd.DataFrame({'Temperature' : Temperature, 'Derivative' : Derivatives})
Tm_row = DF_derivatives[DF_derivatives['Derivative'] == max(DF_derivatives['Derivative'])]
TmV1= float(Tm_row['Temperature'])
print('The melting temperature is ' + str(TmV1))
```
